# Ear wound regeneration in the African spiny mouse *Acomys cahirinus*

**DOI:** 10.1002/reg2.50

**Published:** 2016-03-09

**Authors:** Dino Matias Santos, Ana Martins Rita, Ignasi Casanellas, Adélia Brito Ova, Inês Maria Araújo, Deborah Power, Gustavo Tiscornia

**Affiliations:** ^1^ Regenerative Medicine Program, Department of Biomedical Sciences and MedicineUniversity of Algarve8005‐139 FaroPortugal; ^2^ Center for Biomedical Research, CBMRUniversity of Algarve8005‐139 FaroPortugal; ^3^ Centro de Ciências do Mar (CCMAR)University of Algarve8005‐139 FaroPortugal

**Keywords:** Acomys, blastema, muscle regeneration, regeneration, spiny mouse

## Abstract

While regeneration occurs in a number of taxonomic groups across the Metazoa, there are very few reports of regeneration in mammals, which generally respond to wounding with fibrotic scarring rather than regeneration. A recent report described skin shedding, skin regeneration and extensive ear punch closure in two rodent species, *Acomys kempi* and *Acomys percivali*. We examined these striking results by testing the capacity for regeneration of a third species, *Acomys cahirinus*, and found a remarkable capacity to repair full thickness circular punches in the ear pinna. Four‐millimeter‐diameter wounds closed completely in 2 months in 100% of ear punches tested. Histology showed extensive formation of elastic cartilage, adipose tissue, dermis, epidermis and abundant hair follicles in the repaired region. Furthermore, we demonstrated abundant angiogenesis and unequivocal presence of both muscle and nerve fibers in the reconstituted region; in contrast, similar wounds in C57BL/6 mice simply healed the borders of the cut by fibrotic scarring. Our results confirm the regenerative capabilities of *Acomys*, and suggest this model merits further attention.

## Introduction

Adult regeneration, definedas the ability to reconstitute functional tissues and organs in response to damage in adult organisms, occurs in many different groups of metazoans. Almost every phylum includes species capable of regenerating missing body parts, but in every group there is significant variability in regeneration ability (Alvarado 2000). Notable invertebrate ‘regenerators’ include hydra, starfish, annelids and planarians; among vertebrates, regeneration is found in fish, and among amphibians, both in anurans and urodeles (Tanaka 2003; Brockes & Kumar 2005; Slack et al. 2008). In other groups, such as birds, nematodes and cephalochordates, regeneration is in general exceptional or non‐existent (Li et al. 2015).

While the prevailing view is that mammals in general do not show a regenerative response upon wounding (Muneoka et al. 2008), several examples of epimorphic regeneration in mammals have been described. One of the main experimental paradigms for mammalian regeneration is the ear punch assay. The ear pinna is an easily accessible organ composed of a thin central layer of elastic cartilage, dorsal to which there is adipose tissue and a muscle layer, followed by dermis and epidermis with abundant hair follicles and sebaceous glands. Ear structure ventral to the cartilage layer is thinner than the dorsal region, relatively devoid of muscle and adipocytes and shows less abundance of epidermal appendages. An ear punch assay consists of a full thickness (usually circular) wound of variable size in which regeneration or wound healing can be studied. Epimorphic closure of full thickness ear punches in rabbits was first described in 1953 (Williams‐Boyce & Daniel 1986). Since then, a number of reports have made use of this system for research in regeneration, but the underlying cellular and molecular mechanisms remain poorly characterized. Ear wound regeneration has also been reported in cats, pikes and echo‐locating bats (Goss 1980).

Ear punches, traditionally utilized as an identification method in mice, have gradually become less used due not only to ethical concerns and the development of alternative identification methods but also to the recognition that some mouse laboratory strains were capable of a degree of wound closure that could result in altered or ambiguous identification patterns. In 1998, closure of full thickness 2‐mm ear wounds was first reported in the Murphy Roths Large/lymphoproliferative (MRL/MpJ‐Faslpr/J) mouse strain (Clark et al. 1998), an extensively studied model for systemic lupus erythematosus (Theofilopoulos 1993). This phenotype was independent of both the Fas mutation and the autoimmune phenotypes of the MRL strain and also shared by the closely related Large/J strain (Kench et al. 1999). The results gave rise to a number of publications examining the regenerative and healing capabilities of these and other selected strains across a range of tissues and injury types (Heydemann 2012; Rai & Sandell 2014). The regenerative ability of the MRL/MpJ strain has been reported to extend to other tissues, such as skin, cornea, nerve injuries and articular cartilage lesions. Regeneration of cardiac lesions remains somewhat controversial, with a number of groups claiming a degree of cardiac regeneration and several others reporting little or no difference with non‐regenerating mouse strains (Heydemann 2012). In recent years, the study of these strains has begun to suggest a number of biological processes that could underlie this regenerative phenotype. Regeneration correlates with decreased fibrosis and scarring (Ueno et al. 2005) and appears to be mediated by increased proliferation and decreased apoptosis, inhibition of basement membrane formation, changes in extracellular matrix and stimulation of cellular differentiation(Gourevitch et al. 2003; Tucker et al. 2008).

A number of studies have sought to identify the underlying molecular mechanisms and pathways involved in MRL mouse regeneration, not only in ear closure but also in digit tip regeneration and spinal chord injury repair. One report has shown upregulation of genes involved in DNA replication and repair, protein synthesis, activation of catabolic pathways and changes in cell adhesion molecules (Thuret et al. 2012). Other studies have found increased levels of metalloproteinases to be involved in basement membrane degradation (Tucker et al. 2008) or matrix remodeling (Gourevitch et al. 2003), activation of keratin‐specific genes such as *Krt66* and *Krt6* (Cheng et al. 2013) and involvement of the transforming growth factor β, Bmp and Wnt signaling pathways (Chadwick et al. 2007; Rai et al. 2013). Remarkably, a screen for ear punch closure revealed that mutations in the transforming growth factor β type I receptor can make C57Bl/6, a non‐healer strain, able to close 2‐mm wounds (Liu et al. 2011). Enhancement of regeneration by previous wounding has led to the suggestion of a possible role for circulating cytokines (Davis et al. 2005). Furthermore, analysis of cell cycle profiles of fibroblasts in the MRL mouse led to the identification that the cell cycle regulator p21 plays a significant role in the process, and that a p21 knockout non‐healer strain can in fact acquire a healer phenotype (Bedelbaeva et al. 2010). Overexpression of Lin28, an RNA binding protein involved in repression of the microRNA let7, has also been shown to enhance ear closure in non‐healer mouse strains (Shyh‐Chang et al. 2013).

However, despite this progress, mammalian regeneration is far from well understood, and part of the limitation is that the instances of regeneration in mammals remain few. Therefore, any report of a new mammal capable of regeneration would be of great interest.

A recent description of skin shedding and regeneration of skin wounds in two species (*Acomys percivali* and *Acomys kempi*) of a rodent commonly known as the African spiny mouse merits attention due to the strong claims of the report (Seifert et al. 2012). Despite their common name, this genus belongs to the subfamily Deomynae, not Murinae, and is phylogenetically more related to gerbils than to mice (Chevret et al. 1993). These two species show skin autonomy due to weak skin with a propensity to tear, resulting in extensive full thickness wounds that heal quickly with the presence of abundant hair follicles in the wound bed, presumably a trait selected to escape predation. This regenerative phenotype is also found in the animal's ears, whose structure is in general similar to that of *Mus*. Full thickness circular punches 4 mm in diameter closed completely in 2 months, and histological examination showed the regenerated area to contain dermis, epidermis, cartilage, hair follicles and adipose tissue. Notably, muscle was not observed in the regenerated tissue, and the report did not address whether nerve fibers and vasculature were present or not. In contrast, *Mus musculus* (C57BL/6) healed their wounds by simple fibrotic scarring and showed little or no signs of regeneration. Further analysis suggested that regeneration in *A. kempi* and *A. percivali* proceeds through the formation of a structure with blastema‐like traits, including continued proliferation throughout the regenerative process and establishment of a specialized epidermal signaling center at the leading edge of regeneration characterized by loss of epidermal stratification, disorganization of basal keratinocytes and absence of a mature basal lamina. None of these events was detected in *Mus* (Seifert et al. 2012).

In this study, our goal was to test the reproducibility of these ear punch results in a third species of the genus, *Acomys cahirinus*. We performed 4‐mm ear punches and measured closure rate in *Acomys* versus *Mus* (C57BL/6). We found full closure of wounds in *Acomys* but not *Mus* at 2 months in 100% of all wounds tested. Histological examination confirmed the presence of dermis, epidermis, adipose tissue, cartilage and hair follicles in the reconstituted tissue. Furthermore, we observed abundant angiogenesis and unequivocal presence of muscle and nerve fibers in the regenerated area. In summary, our results confirm that *A. cahirinus* is indeed capable of regeneration of 4‐mm ear punch wounds and is worthy of further study.

## Results

### Closure of ear wounds in *Mus* versus *Acomys*

**Figure 1:**
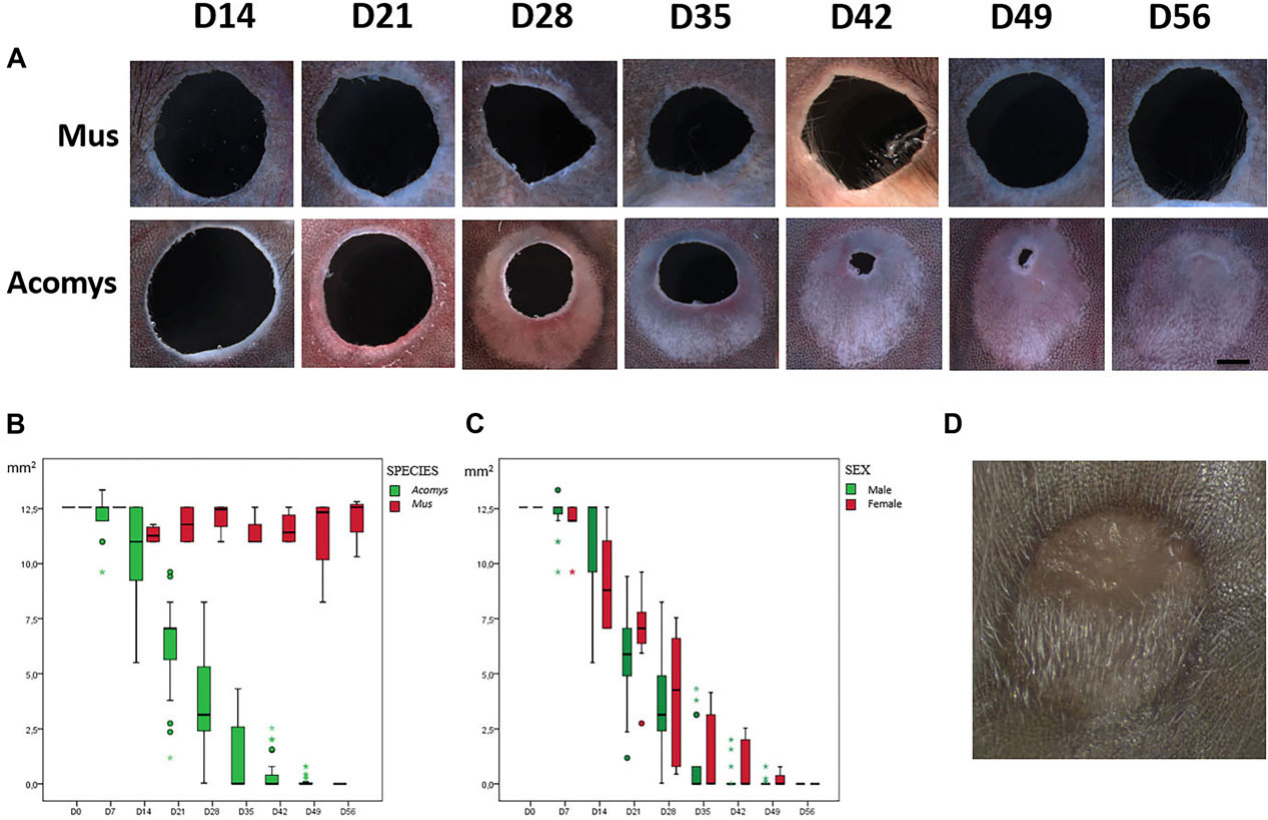
(A) Timeline of ear punch closure of *A. cahirinus* versus *Mus* C57BL/6 (distal−proximal axis shown vertically with distal at top and proximal at bottom for all panels) at weekly intervals between day 14 and day 56 (220×, scale bar 1 mm). (B) Quantification of ear punch area from day 0 to day 56 in *Acomys* (in green) versus *Mus* (in red). (C) Boxplot of ear punch area from day 0 to day 56 in *Acomys* males (in green) versus *Acomys* females (in red). (D) Abundant hair follicles within completely regenerated *A. cahirinus* 4‐mm ear punch.

In order to test the regenerative capacity of *A. cahirinus* in comparison to *M. musculus* (C57BL/6), two independent experiments were performed. A total of 15 adult *Acomys* animals (10 males, five females) were used. Fourteen adult C57BL/6 mice (seven males, seven females) were used as controls. Animals were anesthetized with isofluorane and a 4‐mm circular full thickness punch was performed in each ear. The ratio of ear punch to total ear surface was between 0.3 and 0.4 for all animals, with *Acomys* animals tending towards 0.3 and *Mus* animals tending towards 0.4. The punch was located centrally in the ear pinna, avoiding major ear vessels and resulting in minimal bleeding. The cuts were sharply defined, with minimal damage due to crushing. Animals did not seem overly distressed and engaged in normal feeding and grooming behavior shortly after the procedure. Animals were re‐anesthetized with isofluorane and examined on a weekly basis for up to 2 months. One ear from an *Acomys* animal suffered a tear that disrupted the circular wound and another *Acomys* specimen died (1 and 2 weeks after wounding, respectively); these wounds were eliminated from the analysis. Vertical and transverse measurements of wound diameter were taken and used to calculate wound surface. Behavior of wounds in *M. musculus* was unremarkable, with wound borders showing minimal inflammation and appearing fully healed after 2 weeks. No appreciable closure in wound surface was noted throughout the 2‐month observation period (Fig. 1A). In contrast, wounds in *A. cahirinus* took on a completely different aspect. By day 21, a ring of clear, translucent tissue had formed around the cut and had already begun growing inwards towards the center of the wound (Fig. 1A). This growth continued throughout the observation period until full closure was achieved in 100% of the wounds evaluated by day 56 of the study. The closure curves for both species and between males and females of *Acomys* were quantified and compared using a general linear mixed model, a statistical methodwell suited to longitudinal studies. The wound closure curves between species (Fig. 1B) showed a statistically significant difference (*P* < 0.05), but no difference was observed between male and female animals (Fig. 1C) (*P* = 0.98). Notably, growth was asymmetrical in the proximal−distal axis, with more growth on the proximal side than the distal side. No right−left axis asymmetry was observed. As wound closure progressed, robust formation of new hair follicles was observed in new tissue, appearing first in the proximal region (Fig. 1D).

### Histological characterization

**Figure 2:**
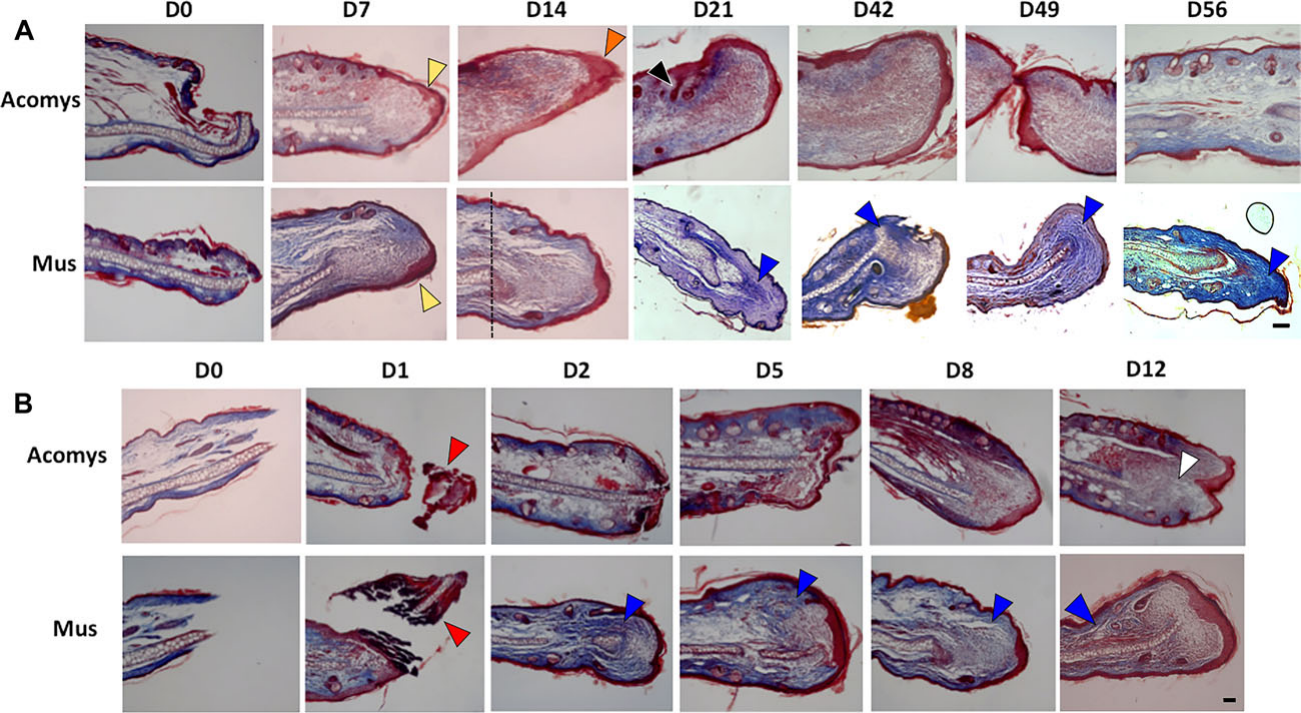
Histological sections of *A. cahirinus* versus *Mus* C57BL/6 stained with Masson's trichrome stain (50×, scale bar 100 μm) at weekly intervals. (A) *A. cahirinus*, day 0 to day 56. Yellow arrowheads show complete epithelialization at day 7; orange arrowhead shows thickened epidermis typically observed at day 14 in *Acomys*; vertical dotted line at day 14 shows original plane of wounding. (B) *Mus* C57BL/6, day 0 to day 12. Red arrowheads show epithelialization and complete sloughing of necrotic tissue by day 2; white arrowhead shows expanding blastema by day 12. In both (A) and (B), blue arrowheads show increased blue coloring indicative of increased collagen deposition in *Mus*.

Two ear samples from representative animals of both *M. musculus* and *A. cahirinus* were taken at weekly intervals, cut serially throughout the wounded region and stained with Masson's trichrome stain. Histological findings were consistent within experimental groups and for a given time point. A representative panel is shown in Fig. 2A. Use of biopsy punches to create the wounds resulted in cleanly cut wound borders with minimal damage. By day 7, re‐epithelialization was complete in both species, below which a mass of loosely packed tissue was observed. By day 14, histological differences between the two species had become apparent. The distance between the plane of the original cut (identified by the presence of mature cartilage) and the border of the wound was noticeably greater for *Acomys* than for C57BL/6. In both species, a variable degree of thickening of the wound epithelium was observed but was in general more pronounced for *Acomys* than for C57BL/6 and was particularly noticeable around day 14. A more intense blue‐colored staining was observed in C57BL/6 samples at every time point, suggesting that collagen deposition started earlier and reached higher levels in *Mus* but not *Acomys*.

By day 21, formation of new hair follicles was clearly observable beyond the plane of wounding in *Acomys*. Epidermal ‘downgrowths’ extending into the sub‐epidermal (blastema‐like) area have been reported in the literature (Rajnoch et al. 2003) but were not observed consistently in our study. In sum, the differences in the regenerative versus scarring response between the two species were striking over the 2‐month observation period, with complete closure of 4‐mm full thickness wounds in 100% of ears injured in *Acomys* but virtually no closure in C57BL/6.

Since growth rate and histological differences were apparent within 2 weeks of wounding, we subsequently performed histological analysis at regular time points between day 0 and day 12 (Fig. 2B). At day 1 after wounding, mild local inflammation was evident in both species, but resolved by day 3. Necrosis seemed more pronounced in C57BL/6 compared to *Acomys*. In both species partial or total re‐epithelialization was obvious by day 1 and sloughing off of necrotized tissue was essentially complete by day 2. At the later time points (days 8 and 12), the behavior between species diverged: while *Acomys* showed lower collagen deposition and formed a blastema‐like structure that started to expand, C57BL/6 showed greater collagen deposition and did not give rise to an expanding blastema‐like structure.

**Figure 3:**
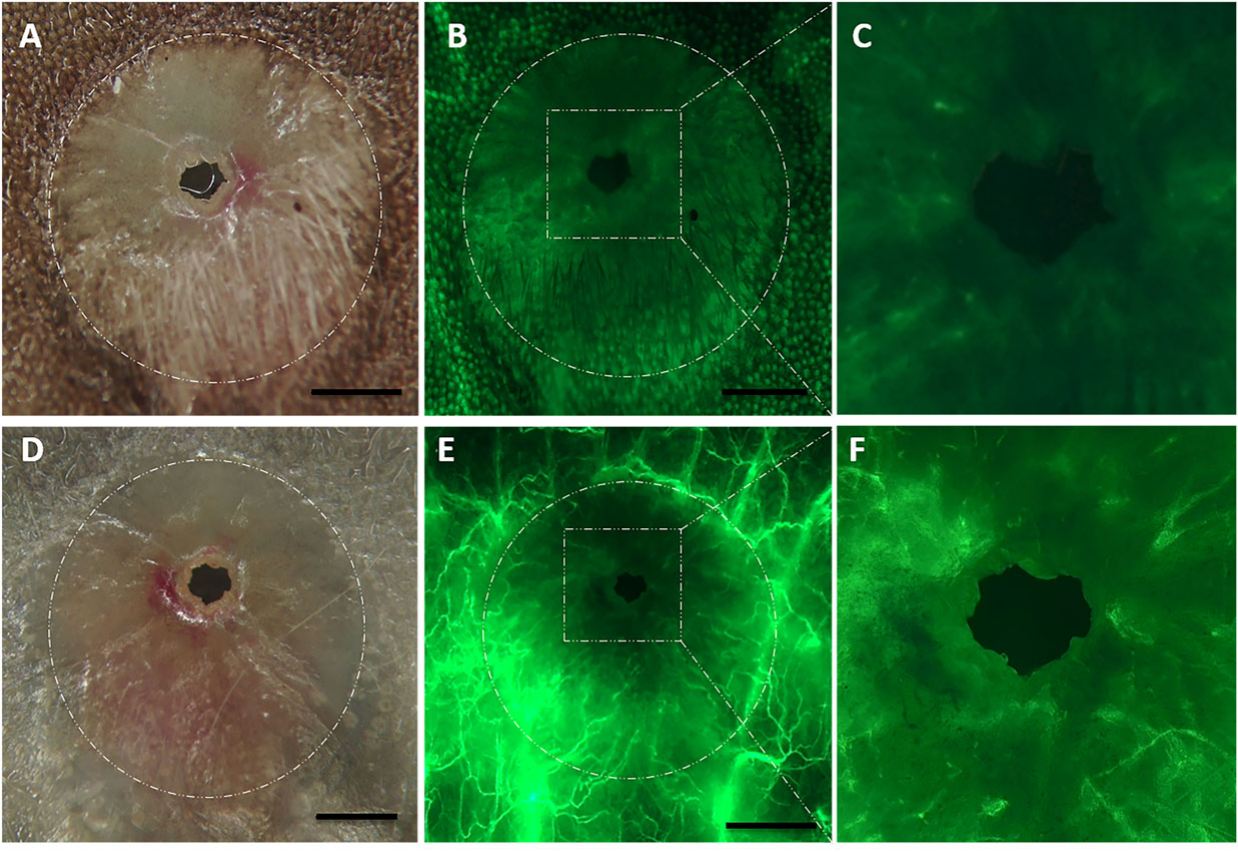
*A. cahirinus* regenerating ear punch at day 49: (A), (B), (C) dorsal view; (D), (E), (F) ventral view. (A), (D) White light (120×, scale bar 1 mm); (B), (E) UV light after fluorescein−dextran injection (120×, scale bar 1 mm); (C), (F) UV light after fluorescein−dextran injection (420×). Circular dotted white line in (A), (B), (E), (F) represents the original border of the wound.

In order to determine the degree of angiogenesis in the newly formed tissue, 7 weeks after wounding, two animals that had almost completed closing their wounds were injected through the left ventricle of the heart with fluorescein labeled dextran as indicated in Methods. Using this method, an abundant and well‐developed capillary network clearly located within the regenerated area was visualized (Fig. 3). The observed network was located on the ventral side of the ear pinna and was more abundant in the posterior region than the anterior region.

**Figure 4:**
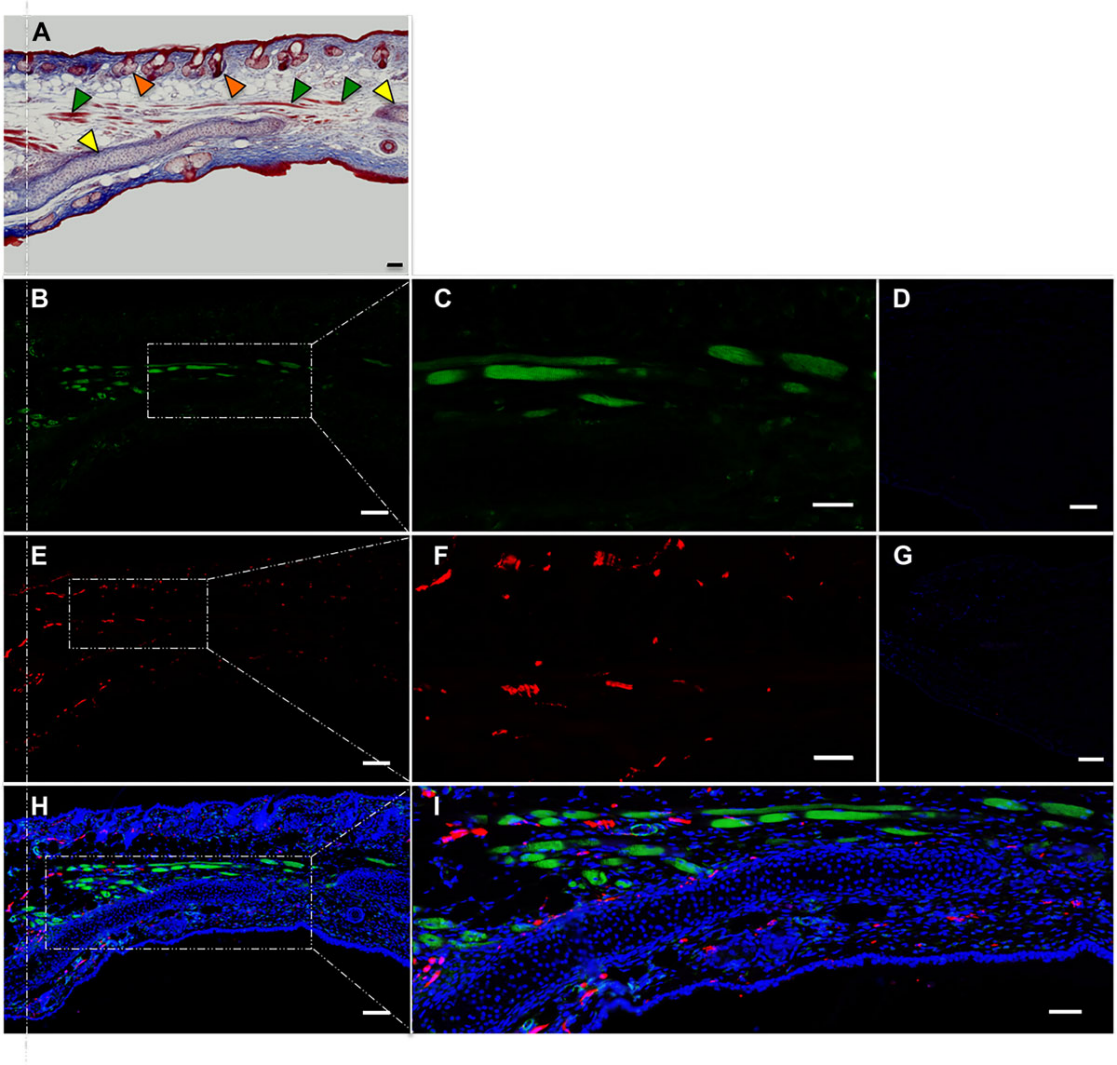
Immunofluorescence analysis of regenerated *A. cahirinus* ear punch. (A) Masson's trichrome stain (50x); vertical dotted line indicates original plane of wounding; yellow arrowheads indicate cartilage; green arrowheads indicate muscle; orange arrowheads indicate hair follicles. (B) Incubated with anti‐actin (50x). (C) Probed with anti‐actin (200×). (D) Negative control in which the primary antiserum was omitted (200×). (E) Incubated with anti‐TUJ1 (50x). (F) Probed with anti‐TUJ1 (200×). (G) Negative control stained with secondary antibody only (200×). (H) Merge of B + E + Dapi (5x). (I) Merge of B + E + Dapi (200×). All scale bars 100 μm.

Histological analysis with Masson's trichrome staining of regenerated tissue at day 56 confirmed re‐establishment of general tissue architecture (Fig. 4A). Elastic cartilage grew extensively into the regenerated area, but was morphologically distinct from the cartilage of uninjured pinna. Chondrocytes were smaller and stained more intensely than the original cartilage, suggesting that cartilage is still immature at this time point (Fig. S1). A well‐developed adipocyte layer formed dorsal to the elastic cartilage. Well defined dermal and epidermal layers with abundant collagen deposition and numerous hair follicles and associated sebaceous glands were also evident. Interestingly, we clearly observed abundant muscle fibers in the regenerated region (Fig. 4A, B, H, I, and Fig. S1). We performed immunofluorescence with an actin antibody and confirmed the presence of actin‐positive myofibers (Fig. 4C). Under high magnification, a typical striated muscle actin banding pattern was evident (Fig. S2). Similarly, immunofluorescence against beta‐tubulin (TUJ1) revealed abundant nerve fibers in the regenerated region (Fig. 4E, F). The regenerated ear of the animals gave no indication of having lost integrity or function; animals could move their ears normally, and sensitivity seemed unaffected, as judged by ear twitching when animals under light anesthesia were mechanically stimulated in the regenerated region.

In order to confirm our results, six independent animals were analyzed by Masson trichrome staining and immunofluorescence 3 months after the ear wound had closed. Interestingly, at this time point, cartilage in the regenerated region had matured into a morphology that was indistinguishable from that of uninjured cartilage (Fig. S3). We confirmed the presence of striatal myofibers with characteristic actin banding pattern in the regenerated region in all animals except one. The amount of regenerated muscle varied between animals. Two animals showed abundant muscle fibers, three animals showed somewhat fewer muscle fibers, and the remaining animal had few or no muscle fibers in the regenerated region. Specificity of the antibodies used was tested by western blot, and both antibodies detected protein bands of the expected size in *Acomys* and *Mus* brain and striated muscle as well as in uninjured and regenerated *Acomys* ear samples (Fig. S4).

## Discussion

Regeneration, defined as injury repair involving re‐establishment of tissue or organ architecture and function in adult individuals of an animal species, has been studied in a number of species in which the trait appears. Among these models are the hydra, annelids, planarian flatworms, fish (mainly the zebrafish, *Danio rerio*) and both anuran and urodel amphibians. While the intrinsic interest of these invertebrate and lower vertebrate regeneration models is beyond doubt, it is notorious that examples of regeneration in higher vertebrates (particularly mammals) are relatively rare.

The main model of mammalian regeneration is the MRL mouse, shown by several research groups to be capable of regenerating a number of tissues and structures. The main experimental system has been closure of ear wounds due to the relative phylogenetic closeness of mice to humans, the well‐established advantages of the mouse model as an experimental system and the easy accessibility of the ear as a target organ for regeneration. Otherwise, regeneration has proved rather exceptional among rodents (Heydemann 2012; Rai & Sandell 2014). In the last two decades the emerging field of regenerative medicine has integrated advances from the areas of stem cell biology, direct reprogramming and tissue engineering. Compounding this approach with a better understanding of the underlying mechanisms that inhibit regeneration in mammals would be of great value, and therefore any novel example of regeneration in mammals is worthy of attention.

Recently, it was reported that a relatively obscure genus, *Acomys*, commonly known as the African spiny mouse, has remarkable regenerative capabilities (Seifert et al. 2012). Surprisingly, 4‐mm circular full thickness wounds in the ear pinna quickly formed a ring of highly proliferative tissue (blastema‐like) that grew inwards towards the wound, closing it completely in 2 months. Histological examination showed that the new tissue was not a fibrotic scar or undifferentiated cells but consisted of tissue and cell types normally found in the ear, namely elastic cartilage, adipose tissue, dermis and epidermis, including abundant hair follicles, organized in an architecture similar to that found in normal, uninjured ear. Muscle was not observed, and the report did not mention whether nerve fibers or vasculature had been observed in the regenerated area.

We sought to test the reproducibility of these results in a third species of the genus, *A. cahirinus*, commonly known as the Egyptian spiny mouse. A direct comparison between *A. cahirinus* and *M. musculus* C57BL/6 using a 4‐mm ear punch assay showed distinct differences between the two groups. Notably, ear punches in the MRL mice reported in the literature have been 2 mm in diameter (Heydemann 2012), with no reports that we are aware of mentioning punches of larger diameter. As our focus was to test whether *A. cahirinus* was capable of regeneration of 4‐mm wounds, we chose a C57Bl/6 as a well known ‘non‐regenerator’ control. While C57BL/6 animals healed the borders of their wounds without any obvious closure of the surface area of the ear opening, 100% of *A. cahirinus* animals evaluated completely closed their openings within the 60‐day study period. This time course reproduces the exact time course of regeneration previously reported (Seifert et al. 2012). The set of *Acomys* animals used in our study included both female and male animals. There have been reports in the literature that healing and regeneration can be influenced by sex (Joseph & Dyson 1965; Grimes & Goss 1970; Plackett et al. 2010). Notably, we found no differences between male and female animals. One punched ear suffered a tear which disrupted the circular shape of the wound, which led to rapid arrest of the ear closure process. While this occurred only in a single instance in this study, we have observed a similar outcome in several other ear‐punched animals of our colony: disrupted ear wounds do not complete the regeneration process. This suggests that successful regeneration depends on a spatial information component, perhaps the establishment of a required morphogenetic signaling field in the proper orientation. Alternatively, it could point to the importance of an intact circulatory system in the surrounding non‐injured regions, possibly by affecting circulation of cytokines, which have been suggested to be involved in ear closure in the MRL mouse (Kench et al. 1999). Histological examination showed that the overall structure of the closed region was remarkably similar to that of uninjured regions of the ear. Elastic cartilage, adipose tissue, dermis and epidermis were clearly observed, including abundant epidermal appendages such as hair follicles and sebaceous glands. Such extensive tissue would not be expected to develop or be sustained in the absence of angiogenesis in the regenerated region. Injection of fluorescein−dextran into the general circulation confirmed that angiogenesis had occurred throughout the reconstituted tissue, albeit more abundantly on the ventral than on the dorsal side of the ear. In contrast to the report by Seifert et al., our histological stain showed the presence of what appeared to be muscle fibers positioned throughout the repaired region in six out of seven animals. Immunofluorescence using an actin antibody confirmed the finding and revealed the typical actin banding pattern for striated muscle under high magnification. It is unclear why Seifert et al. have not observed this tissue, but a degree of genetic variability between animal colonies is a possibility. Similarly, an antibody against βIII‐tubulin revealed the presence of abundant nerve fibers throughout the dermis and epidermis.

Our results confirm that *Acomys* is indeed capable of robust repair of relatively large ear wounds including muscle, vasculature and nerve fibers and is worthy of further study. Quantitative measurement of expression profiles of genes of interest during regeneration will need to await genome and transcriptome sequencing and annotation, as yet unavailable. What are the mechanisms underlying ear wound closure in *A. cahirinus*? The original structure of the wounded organ, based on a continuous surface of relatively non‐elastic cartilage, suggests that contraction plays a minimal role in ear wound closure. The large area reconstituted (12.5 mm^2^) is remarkable and the histological structure confirms that it is indeed the result of a regenerative process, although a contribution by cell migration cannot be ruled out. Whether this regenerative phenomenon involves dedifferentiation of mature cells to less restricted cells capable of subsequent proliferation and re‐differentiation or involves mobilization of a stem cell compartment remains to be elucidated. The finding of ear regeneration in *A. cahirinus* in addition to *A. kempi* and *A. percivali* suggests this may be a genus‐wide trait, and perhaps found in other genera of the subfamily Deomynae or, further, in other subfamilies of the Muridae. Interesting questions for future research are what are the cellular and molecular mechanisms underlying this trait, whether the mechanisms involved in skin regeneration and ear regeneration are related or not, if other tissues and organs of *Acomys* are capable of regeneration, and whether non‐regenerating mammals could be induced to regenerate.

## Methods

### Animal procedures

Specimens of *A. cahirinus* wereacquired from a local breeder and kept at the animal facility at the University of Algarve. Species was confirmed by barcoding of the mitochondrial CoxI gene using universal primers (FP 5′‐TGTAAAACGACGGCCAGTTCTCAACCAACCACAA‐AGACATTGG‐3′ and RP 5′‐CAGGAAACAGCTATGACT‐AGACTTCTGGGTGGCCAAAGAATCA‐3′) (Ivanova et al. 2007). The resulting 684‐bp sequence was imputed into Genbank and a >99% identity to *A. cahirinus* was obtained. Animals were fed a diet of mouse chow (Mucedola 4RF25/A, Milano, Italy) and assorted seeds (commercial bird feed) in a 1:1 ratio, supplemented with fresh apple or carrots weekly. *M. musculus* (C57BL/6) were provided by the animal facility at the University of Algarve and fed mouse chow. Both species were kept on a 13/11 h light/dark cycle. The study was approved by the Ethical Committee of the Centro de Biomedicina Molecular e Estrutural (CBME‐EC‐0006). Experiments were conducted following national and European guidelines, and all measures were taken to minimize discomfort and the number of animals used in the study. Animals were anesthetized with isofluorane (Isoflo, Abbott Laboratories, Berkshire, UK), the ears were disinfected with 70% EtOH and circular punches 4 mm in diameter were made in the center of the ear pinna using biopsy punches (Miltex #33‐44, 4 mm, Integralife Sciences, York, USA). Vertical and horizontal measurements of wound diameter were taken weekly and used to calculate punch surface. Ear wound surface measurements over time for *Acomys* and *Mus*, and for male and female *Acomys*, were analyzed with a general linear mixed model. The Statistical Package for the Social Sciences (SPSS) 22.0 was used for the analysis. To assess the results obtained, a *P* value <0.05 was set as statistically significant. At regular intervals representative animals were anesthetized with xylazine (Rompun 2%, KVP Pharma, Kiel, Germany)/ketamine (Imalgene 1000, Lyon, France) (1 mg/mL xylazine, 10 mg/mL ketamine) by intraperitoneal injection at a rate of 0.1 mL/10 g of weight until animals were deemed unresponsive to footpad pinching, and the chosen ear was harvested, photographed and subjected to histological and immunofluorescence analysis.

### Histology and immunofluorescence analysis

Samples were processed using standard histological protocols. Briefly, samples were fixed in methanol, dehydrated, and embedded in paraffin wax. Transverse sections (10 μm thick) were prepared and mounted on poly‐l‐lysine coated slides. For Masson's trichrome stain a commercial kit was used (Bio‐Optica 04‐010802, Milan, Italy) on dewaxed and rehydrated sections. For immunofluorescence, dewaxed and rehydrated section tissues were subjected to antigen retrieval using 20 × 5 sec microwave pulses in sodium citrate (0.1 mol/L, pH 6), and blocked with 2% donkey serum. Primary antibodies used in this study were goat anti‐actin (sc‐1616, 1/250 dilution, Santa Cruz Biotechnologies, Dallas, TX, USA) and mouse anti‐β‐tubulin (TUJ‐1 clone, MMS‐435‐250, 1/1000 dilution, Covance, Burlington, NC, USA). Secondary antibodies used were donkey anti‐goat IgG (H+L) secondary antibody, Alexa Fluor® 488 conjugate (A‐11055, Life Technologies, Waltham, MA, USA), and donkey anti‐mouse IgG (H+L) secondary antibody, Alexa Fluor® 594 conjugate (Life Technologies, A‐21203).

### Visualization of vasculature

Ten weeks after ear punching, representative animals with remaining wounds approximately 0.5 mm in diameter were put under deep anesthesia with xylazine/ketamine and perfused with 200 mL phosphate buffered saline 1×, followed by 40 mL of a fluorescein isothiocyanate−dextran solution (FD2000S, 50 mg/ml, Sigma‐Aldrich, St Louis, MO, USA) via intraventricular injection with a peristaltic pump. Ears were harvested and observed immediately.

## Supporting information

Additional Supporting Information may be found in the online version of this article at the publisher's website:

**Supplementary Figure S1**: A) Masson's Trichrome stain of *Acomys* ear 56 days after wounding (50x, scale bar 100 um). Vertical dashed line represents original plane of wounding, separating uninjured region (posterior), from regenerated region (anterior). White arrowheads show hair follicles; yellow arrowheads show muscle fibers; orange arrowheads show adipocytes; blue arrowheads show elastic cartilage. B) Uninjured cartilage (630x, scale bar 10 um). C) Regenerated cartilage (630x scale bar 10 um).
**Supplementary Figure S2**: Immunofluorescence with an anti‐actin antibody on regenerated Acomys ear 56 days after wounding (400x, scale bar 20 um).
**Supplementary Figure S3**: A) Hematoxylin‐eosin stain of *Acomys* ear 3 months after wound closure (50x, scale bar 100 um). Image shows regenerated region. Yellow arrowheads show muscle fibers; orange arrowheads show adipocytes; blue arrowheads show elastic cartilage. B and C) Morphology of regenerated cartilage (200x, scale bar 20 um).
**Supplementary Figure S4**: Muscle and neuronal markers are found in the regenerated tissue of *A. cahirinus* ear: 5 μg of brain protein extract, 50 μg of femoral muscle of *A. cahirinus* or Mus C57BL/6, and 50 ?g of non‐injured ear (NE) or regenerated *A. cahirinus* ear (RE) protein extract were run (10% PAGE), transferred and incubated with anti‐actin (1/500) or anti‐TUJ1 (1/1000).
