# Supplementary figures and images for: Ear wound regeneration in the African spiny mouse Acomys cahirinus
Source: Regeneration (Oxf). 2016 Mar 9;3(1):52–61. doi: 10.1002/reg2.50 (PMC4857749; doi:10.1002/reg2.50)

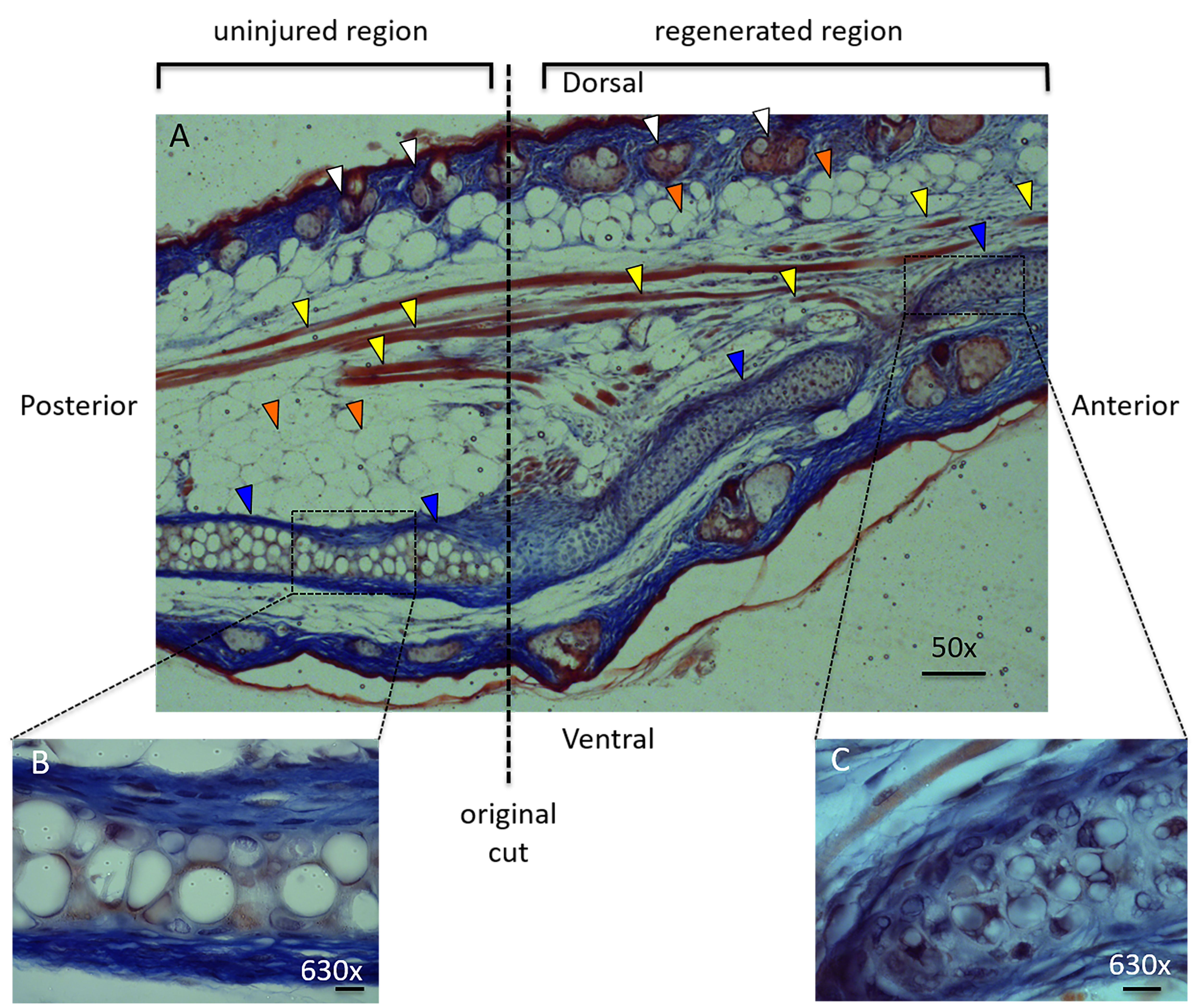

Supplement: Supplementary file 1 — Supplementary Figure S1: A) Masson's Trichrome stain of Acomys ear 56 days after wounding (50x, scale bar 100 um). Vertical dashed line represents original plane of wounding, separating uninjured region (posterior), from regenerated region (anterior). White arrowheads show hair follicles; yellow arrowheads show muscle fibers; orange arrowheads show adipocytes; blue arrowheads show elastic cartilage. B) Uninjured cartilage (630x, scale bar 10 um). C) Regenerated cartilage (630x scale bar 10 um). Supplementary Figure S2: Immunofluorescence with an anti‐actin antibody on regenerated Acomys ear 56 days after wounding (400x, scale bar 20 um). Supplementary Figure S3: A) Hematoxylin‐eosin stain of Acomys ear 3 months after wound closure (50x, scale bar 100 um). Image shows regenerated region. Yellow arrowheads show muscle fibers; orange arrowheads show adipocytes; blue arrowheads show elastic cartilage. B and C) Morphology of regenerated cartilage (200x, scale bar 20 um). Supplementary Figure S4: Muscle and neuronal markers are found in the regenerated tissue of A. cahirinus ear: 5 μg of brain protein extract, 50 μg of femoral muscle of A. cahirinus or Mus C57BL/6, and 50 ?g of non‐injured ear (NE) or regenerated A. cahirinus ear (RE) protein extract were run (10% PAGE), transferred and incubated with anti‐actin (1/500) or anti‐TUJ1 (1/1000). [file REG2-3-52-s001.zip › reg250-sup-0001-FigS1.jpg]

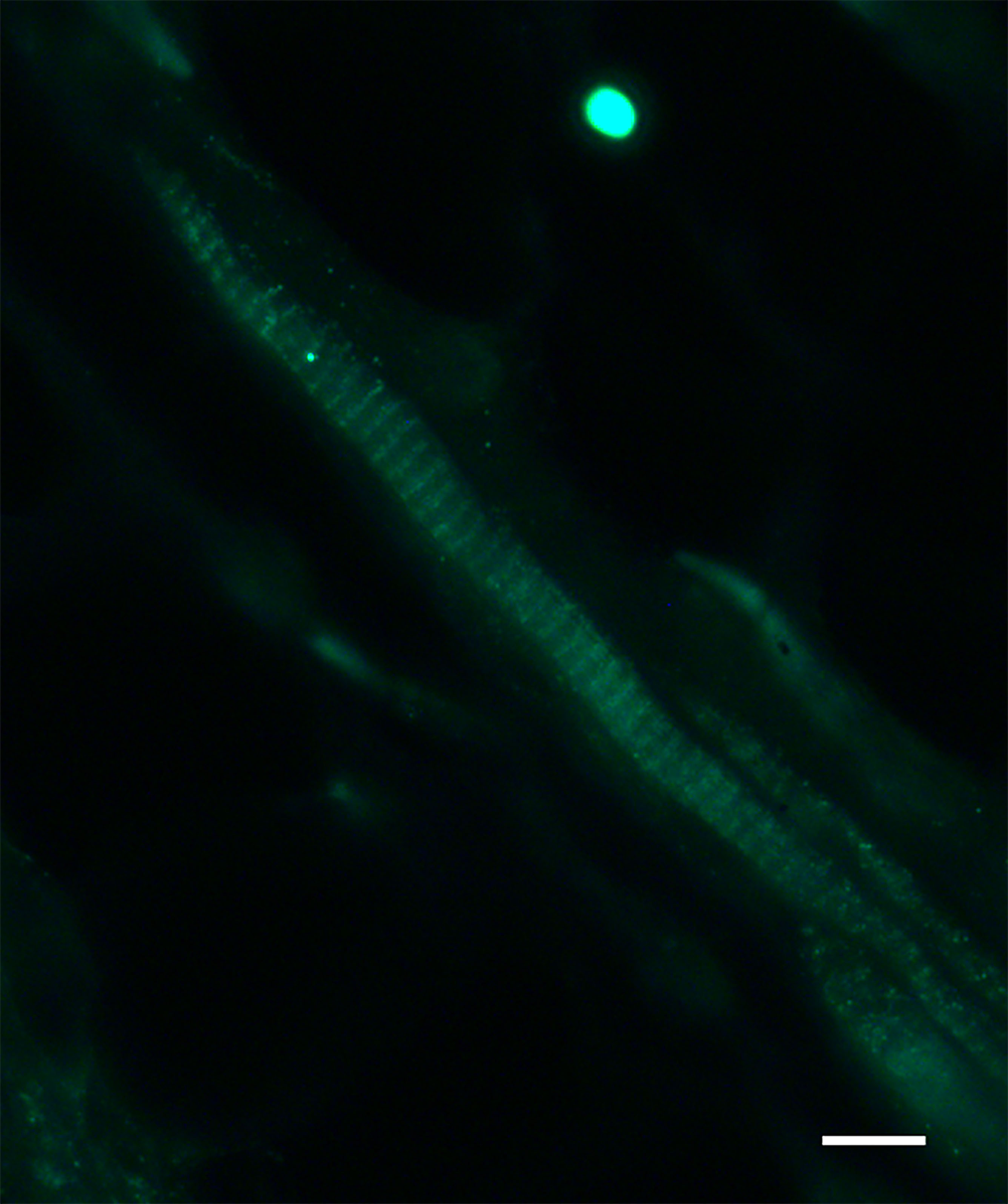

Supplement: Supplementary file 1 — Supplementary Figure S1: A) Masson's Trichrome stain of Acomys ear 56 days after wounding (50x, scale bar 100 um). Vertical dashed line represents original plane of wounding, separating uninjured region (posterior), from regenerated region (anterior). White arrowheads show hair follicles; yellow arrowheads show muscle fibers; orange arrowheads show adipocytes; blue arrowheads show elastic cartilage. B) Uninjured cartilage (630x, scale bar 10 um). C) Regenerated cartilage (630x scale bar 10 um). Supplementary Figure S2: Immunofluorescence with an anti‐actin antibody on regenerated Acomys ear 56 days after wounding (400x, scale bar 20 um). Supplementary Figure S3: A) Hematoxylin‐eosin stain of Acomys ear 3 months after wound closure (50x, scale bar 100 um). Image shows regenerated region. Yellow arrowheads show muscle fibers; orange arrowheads show adipocytes; blue arrowheads show elastic cartilage. B and C) Morphology of regenerated cartilage (200x, scale bar 20 um). Supplementary Figure S4: Muscle and neuronal markers are found in the regenerated tissue of A. cahirinus ear: 5 μg of brain protein extract, 50 μg of femoral muscle of A. cahirinus or Mus C57BL/6, and 50 ?g of non‐injured ear (NE) or regenerated A. cahirinus ear (RE) protein extract were run (10% PAGE), transferred and incubated with anti‐actin (1/500) or anti‐TUJ1 (1/1000). [file REG2-3-52-s001.zip › reg250-sup-0002-FigS2.jpg]

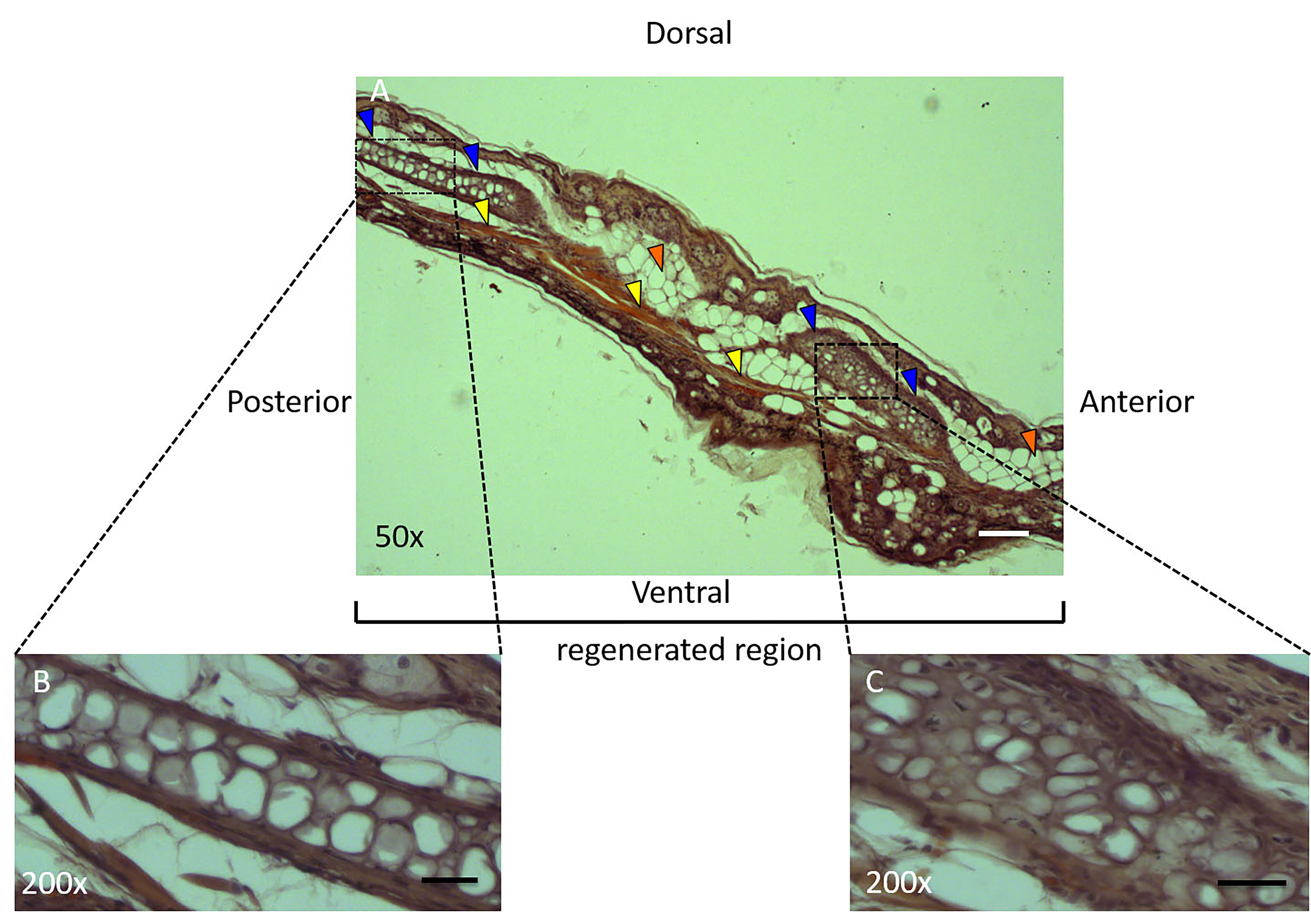

Supplement: Supplementary file 1 — Supplementary Figure S1: A) Masson's Trichrome stain of Acomys ear 56 days after wounding (50x, scale bar 100 um). Vertical dashed line represents original plane of wounding, separating uninjured region (posterior), from regenerated region (anterior). White arrowheads show hair follicles; yellow arrowheads show muscle fibers; orange arrowheads show adipocytes; blue arrowheads show elastic cartilage. B) Uninjured cartilage (630x, scale bar 10 um). C) Regenerated cartilage (630x scale bar 10 um). Supplementary Figure S2: Immunofluorescence with an anti‐actin antibody on regenerated Acomys ear 56 days after wounding (400x, scale bar 20 um). Supplementary Figure S3: A) Hematoxylin‐eosin stain of Acomys ear 3 months after wound closure (50x, scale bar 100 um). Image shows regenerated region. Yellow arrowheads show muscle fibers; orange arrowheads show adipocytes; blue arrowheads show elastic cartilage. B and C) Morphology of regenerated cartilage (200x, scale bar 20 um). Supplementary Figure S4: Muscle and neuronal markers are found in the regenerated tissue of A. cahirinus ear: 5 μg of brain protein extract, 50 μg of femoral muscle of A. cahirinus or Mus C57BL/6, and 50 ?g of non‐injured ear (NE) or regenerated A. cahirinus ear (RE) protein extract were run (10% PAGE), transferred and incubated with anti‐actin (1/500) or anti‐TUJ1 (1/1000). [file REG2-3-52-s001.zip › reg250-sup-0003-FigS3.jpg]

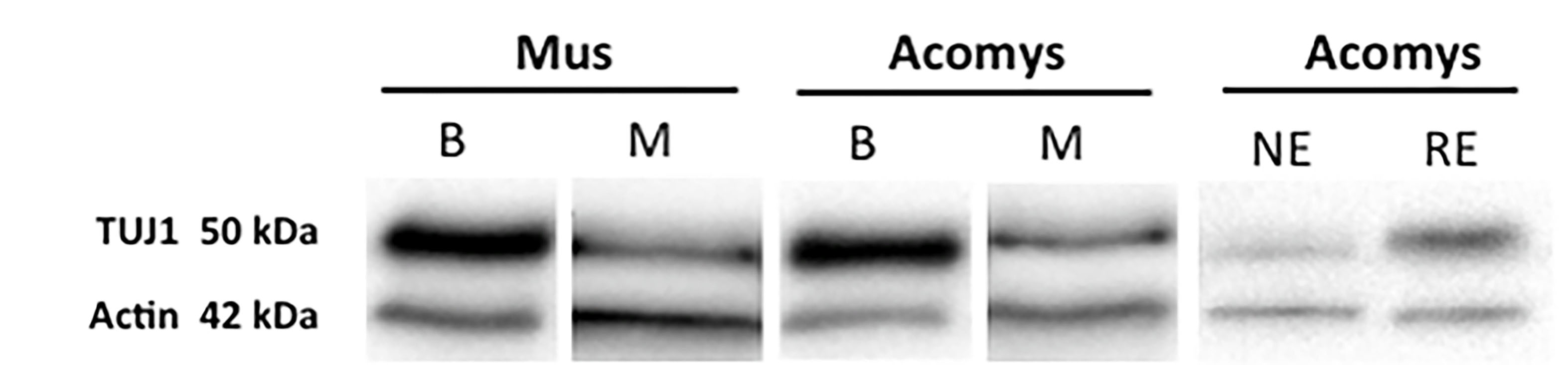

Supplement: Supplementary file 1 — Supplementary Figure S1: A) Masson's Trichrome stain of Acomys ear 56 days after wounding (50x, scale bar 100 um). Vertical dashed line represents original plane of wounding, separating uninjured region (posterior), from regenerated region (anterior). White arrowheads show hair follicles; yellow arrowheads show muscle fibers; orange arrowheads show adipocytes; blue arrowheads show elastic cartilage. B) Uninjured cartilage (630x, scale bar 10 um). C) Regenerated cartilage (630x scale bar 10 um). Supplementary Figure S2: Immunofluorescence with an anti‐actin antibody on regenerated Acomys ear 56 days after wounding (400x, scale bar 20 um). Supplementary Figure S3: A) Hematoxylin‐eosin stain of Acomys ear 3 months after wound closure (50x, scale bar 100 um). Image shows regenerated region. Yellow arrowheads show muscle fibers; orange arrowheads show adipocytes; blue arrowheads show elastic cartilage. B and C) Morphology of regenerated cartilage (200x, scale bar 20 um). Supplementary Figure S4: Muscle and neuronal markers are found in the regenerated tissue of A. cahirinus ear: 5 μg of brain protein extract, 50 μg of femoral muscle of A. cahirinus or Mus C57BL/6, and 50 ?g of non‐injured ear (NE) or regenerated A. cahirinus ear (RE) protein extract were run (10% PAGE), transferred and incubated with anti‐actin (1/500) or anti‐TUJ1 (1/1000). [file REG2-3-52-s001.zip › reg250-sup-0004-FigS4.jpg]
